# Molecular imprinting of glycoproteins: From preparation to cancer theranostics

**DOI:** 10.7150/thno.69189

**Published:** 2022-02-28

**Authors:** Muhammad Mujahid Ali, Shoujun Zhu, Farrukh Raza Amin, Dilshad Hussain, Zhenxia Du, Lianghai Hu

**Affiliations:** 1 Center for Supramolecular Chemical Biology, State Key Laboratory of Supramolecular Structure and Materials, School of Life Sciences, Jilin University, Changchun 130023, China.; 2 State Key Laboratory of Bioelectronics, National Demonstration Center for Experimental Biomedical Engineering Education, Southeast University, Nanjing 210096, China.; 3 Center for Climate Research and Development COMSATS University Islamabad, Park Road, Tarlai Kalan, 45550, Islamabad, Pakistan.; 4 HEJ Research Institute of Chemistry, International Center for Chemical and Biological Sciences, University of Karachi, Karachi-75270, Pakistan.; 5 College of Chemistry, Beijing Key Laboratory of Environmentally Harmful Chemical Analysis, Beijing University of Chemical Technology, Beijing, 100029, China.

**Keywords:** Molecularly imprinting, Glycoproteins, Biosensing, Cancer theranostics, Proteomics

## Abstract

Glycoprotein imprinted polymers have rapidly grown as excellent receptors for cancer targeting, diagnostics, inhibition, and nanomedicines as they specifically target glycans and glycosites overexpressed in various tumors. Compared to natural antibodies, they are easy to synthesize, stable, and cost-efficient. Currently, no study specifically discusses glycoproteins imprinting strategies for cancer theranostics. In this review, firstly we explored various factors involved in designing and synthesis of glycoprotein imprinted materials, including, the characteristics and choice of monomers for imprinting, types of templates and their interactions involved, and the imprinting methods. Secondly, the integration of these MIPs with different probes that have been applied for *in vitro* and *in vivo* targeting for cancer diagnostics including biosensing and bioimaging, and image-guided therapeutic applications as nanomedicines. These Glycoprotein imprinted polymers have been found to specifically target the glycoprotein biomarkers and glycosylated cell receptors overexpressed in different cancers and have been reported as excellent diagnostic tools. As nanomedicines, they have been potentially employed in various modes of cancer therapy such as targeted drug delivery, photodynamic therapy, photothermal therapy, and nanoMIPs themselves as therapeutics for locally killing tumor cells. Although the research is still in its early stages and no real-world clinical trials on humans have been conducted, nanoMIPs have a promising future in this field. We believe these findings will pave the way for MIPs in advanced diagnostics, antibody treatment, and immunotherapy as future nanomedicine for real-world cancer theranostics.

## Introduction

Glycoproteins perform vital roles in several physiological systems, such as molecular recognition, inter-and intra-cell signaling, immune response, sperm-egg interaction, and developmental control. The role of multiple glycoproteins, such as mucins, surface receptors, adhesive proteins, proteoglycans, their transcription factors, and binding ligands is influenced by irregular transcription of sugar moieties, leading to an increase in pancreatic cancer severity and a cancer-friendly microenvironment 1-3. Glycoprotein identification, targeting, and quantification are crucial in life sciences and medical research, clinical diagnostics, medical instruments, and aging 4-7. The progression of malignancies is linked to aberrant structural alterations and improper expressions. As a result, several glycoproteins, glycans, and monosaccharides are frequently utilized as biomarkers for disease diagnosis and treatment. Nevertheless, substantial challenges limit their detection and targeting, especially selective identification of glycoprotein and glycans structural properties 8-11. In addition, glycoproteins have a low concentration in complex biological samples and co-exist with cellular substances, non-glycoproteins, peptides, and small molecules. Consequently, glycoprotein targeting, isolation, purification, and quantification are substantially hindered by the complex matrix. Therefore, it is indeed critical to selectively and specifically recognize the glycosylated molecules for accurate diagnostic and therapeutic applications.

Glycoprotein-induced antibodies are unique in recognizing protein units regardless of specific glycan structures 12. However, they are constrained in their capacity to identify a complete glycan structure. Besides, lectins serve as affinity tools for glycans but cannot deliver structural details about the glycoprotein's fragments 13. Furthermore, the generation of highly efficient antibodies is expensive, tedious, and challenging. Often, antibodies' stability and reproducibility are troublesome 14, 15. Recognizing these shortcomings in biological affinity tools, there is a considerable opportunity and potential for the development of synthetic affinity tools, skilled in exceptionally accurate molecular targeting for specific challenging biological macromolecules 16.

A great challenge in molecular imprinting includes a method of template-induced creation of molecular cavities with identification sites in a substance that arises as a very interesting idea 17. Molecularly imprinted polymers (MIPs) are developed employing several interactions such as covalent bonding, hydrogen bonding, van der Waals, hydrophobic and electrostatic interactions in different monomers and crosslinkers. These monomers are engineered to build recognition sites in imprinted nanoscale cavities complementary template molecules, compatible with shape, dimensions, and functionalities, indicating a vital role in supporting target affinity and selectivity 18, 19. They are simple to synthesize, cost-effective, durable, and stable in a broad pH range, a variety of solvents, and thermal conditions 20. Moreover, in a broad range of analytical tools including chromatographic columns or microextraction systems, MIPs can be conveniently integrated. MIPs have been commonly used as affinity materials for sample pretreatment. Recently, employing MIPs before mass spectrometry, electrochemical techniques 21, surface plasmon resonance 22, surface-enhanced resonance Raman scattering 23, 24, colorimetry 25, and fluorescence 26, have gained increasing interest. In addition, these glycoprotein imprinted nanoparticles have also been potentially applied in cancer diagnostics and treatment 27. Based on their capability to capture covalently and reversibly with cis-diols of glycosylated molecules, boronic acid has appeared as a front-runner to serve as engineered receptors in imprinted cavities for targeting glycoprotein and glycosites that are overexpressed in most malignancies. This unique mechanism enables boronic acids as interesting ligands for a lot of applications targeting specific cancer sites, drug delivery, biosensing, isolation, and self-assembly 16, 28, 29.

In recent years, several reviews have been reported on the application of molecularly imprinted polymers in separation 30, sensing 31, microbe-analysis 32, and cell detection 32. Despite a large number of studies, no review specifically discusses the molecular imprinting strategies for glycoproteins and their applications in sensing glycoprotein cancer markers, tumor imaging, and treatment. In this review, we discussed rational designing regarding the selection of the polymer matrix, templates, immobilizing and capturing mechanisms and some novel synthetic approaches for glycoprotein imprinting. Particularly, integration of these MIPs with sensing probes and tumor drugs explored for *in vitro* and *in vivo* targeting, cancer diagnostics, bioimaging, and therapeutic applications. The existing challenges and prospects of glycoprotein imprinted technology for targeted drug delivery, bioimaging, and clinical cancer biosensing are also discussed.

## 2. Designing and synthesis of MIPs

### 2.1 Brief comparison of different molecularly imprinted materials for glycoprotein

Different molecularly imprinted materials applied for glycoproteins have specific characteristics, their properties are tunable and there are several monomers available. Proper combination and orientation of monomers may lead to enhanced affinity and high selectivity for the target glycoproteins. Some commonly available monomers and crosslinkers are summarized in Table 1.

Comparing these molecularly imprinted materials for glycoproteins, several observations can be made; 1) Facile synthesis is an important parameter to choose the materials for imprinting and in the reported materials, silica 47, polydopamine 47, 48 and polyether sulphone 49 are more facile to prepare as no harsh conditions are required; 2) Easy removal of template molecule can be achieved by time-controlled thickness or by smart imprinted materials. Time-controlled thickness is especially important for surface imprinting because the template is usually buried inside the polymer layer in case of the uncontrolled thickness of the imprinting layer. The thickness of silica 50-52 and polydopamine 47 imprinting layer can be tuned by controlling the reaction time. Smart polymer materials such as poly (N-isopropylacrylamide), whose imprinting cavity channels can be opened by switching the temperature or pH of the solution, allowing the easy removal of the template molecule; 3) Availability of the multifunctional monomers is also very important for high specificity and strong binding, and numerous monomers of silica and acrylate with special functionality are commercially available as described in Table 1; 4) So far, compatibility with boronate affinity, silica, and polydopamine imprinting layer is developed at pH (8.5) suitable for the binding of boronate affinity 45, 48, 53. Considering the various properties of these materials, the preference of which polymer should be used is determined by the particular use. Some of the important features of these materials are compared in Table 2.

### 2.2 Types of Templates and their characteristics to develop MIPs

The application of protein imprinted materials as artificial antibodies has increased significantly in the last decade due to their captivating features such as tolerance to severe conditions, storage durability, and reusability 54. Template molecules are also crucial for glycoprotein imprinted polymer materials. Conventionally, native glycoproteins were used as templates and this simple methodology did not require any special procedure to prepare the template and usually results in appropriate binding capacity and high specificity. However, this approach has apparent drawbacks if native proteins are used as templates 17. First, while ensuring the native conformation of template proteins, the available polymerization methods are limited. Second, acquiring a large amount of high-purity template protein, especially for low-abundance proteins, is challenging. Third, several smaller non-targets bind comparatively large imprinted sites, leading to lower selectivity. To address these issues, some modern technologies are introduced where synthetic receptors of the target protein 55, 56 such as glycan, a monosaccharide, an oligosaccharide 57, or an epitope 58 are used as templates. These strategies offer a more versatile template selection, allowing the imprinting of uncommon, inaccessible, and unstable target glycoproteins. In addition, these segments as templates are small in size and easily removed from the reaction system. However, binding affinity becomes a key challenge in this approach, and larger template coverage is required. Bie et al. 59 used purified glycans for glycan-oriented surface imprinting, obtained by the digestion of target proteins using glycosidase enzyme. This methodology is used as an efficient tool for the precise enrichment of target glycoproteins and their characteristic fragments. These types of MIPs are highly consistent with MS-analysis and structural characterization because they easily release the captured target analyte 60. However, this strategy is not appropriate for the imprinting of long-chain glycopeptides and even cannot be applied to the O-glycans.

**Figure 1:**
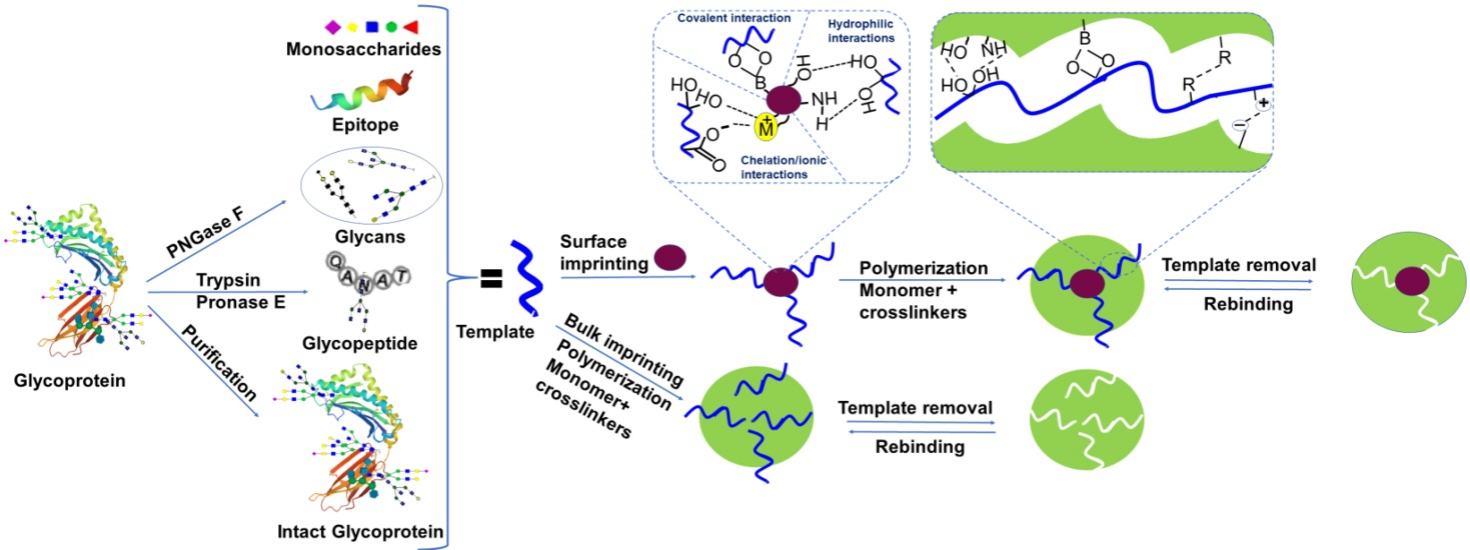
Schematic overview of templates used in glycoproteomics, molecularly imprinted polymer synthesis, and types of interactions involved.

Another approach called *in situ* dual enzymatic digestion is also recommended to fabricate the desired length of glycopeptides. In this methodology, the native glycoprotein carrying the required glycans is immobilized via boronate affinity of the substrate, followed by the *in situ* enzymatic digestion to get glycopeptides of the desired length. Then, a precise imprinting layer is developed and the template is removed by acidic solution. This strategy eliminates tedious fabrication steps template purification and is useful for imprinting both N-glycans and O-glycans 61. The same group also used glycoproteins as a semi template in the organic phase, allowing the imprinting of glycans to control the hydrolysis of silica and consequently thickness of the imprinting layer 62. However, these approaches are not ideal for imprinting glycoproteins, glycans, and monosaccharides without free cis-diols, such as glycosaminoglycans. In such cases, a specific recognizable part of the protein, called epitope is used as a template for the specific glycoprotein. Epitope templates are readily synthesized via artificial fabrication, including rare glycoproteins. In comparison, since they mimic the antibody's recognition domain, epitope-imprinted cavities are more analogous to natural recognition sites, contributing to higher specificity against the target 58. Inspired by the recognition of the 3-dimensional shape of epitopes by antibodies, instead of a linear shape, some studies also used the conformational peptide as a template to get more specificity 63. However, there are also some disadvantages of epitope imprinting. In certain techniques, epitopes are fixed on substrates before imprinting through a functional group such as -SH 64 and -NH_2_ 65 present on the terminal sites. Nevertheless, the coexistence of the same functional groups with other amino acids also contributes to an unfavorable epitope arrangement on the substrate. Epitopes are not immobilized on a substrate in some methods, and the imprinting direction is random, resulting in poor imprinting performance because a major proportion of templates are concealed inside the polymer. Certain approaches are only applicable to restricted situations, and the imprinting of multiple epitopes typically involves tedious conditional selection. Monosaccharides and disaccharides as template molecules have also been reported 66, 67. Monosaccharide templates are stable, easily available, and monosaccharide imprinted materials revealed a high binding capacity, however, they offer poor selectivity. The template preparation approaches have been depicted in Figure 1.

### 2.3 Interaction of template molecules with molecularly imprinted materials

There are two main types of interactions in the imprinting of glycoproteins, covalent and noncovalent, between the template and the specific monomers employed for the production of molecularly imprinted materials (Figure 1). For covalent interactions, boronic acid ligands bind covalently in a reversible manner to form cyclic esters of 1,2, or 1,3-cis-diols in an alkaline environment (typically the pKa < solution pH). This covalent linkage is broken when pH is switched to an acidic environment, thus the selectivity of the recognition sites is typically very high. Furthermore, several novel boronate affinity ligands have been reported, with lower pKa values which can bind in physiological as well as in a slightly acidic environment with strong binding affinity 48. This strong binding affinity enables boronate affinity materials to selectively enrich trace glycosylated biomolecules. Such chemistry brings some important advantages to boronic acid-functionalized materials, including comparatively stronger affinity (as opposed to non-specific bindings), broad-spectrum selectivity (a ligand can bind a wide range of molecules), pH-dependent capture and release, and rapid adsorption/desorption kinetics. Since slightly acidic conditions may release the target compounds from the specimen well, separation based on boronate affinity and sample pretreatment is very consistent with x-omics analysis based on mass spectrometry (MS), where an acidic medium is used for ionization. However, three obvious limitations are associated with traditional boronate affinity materials; (i) non-biocompatible binding pH, (ii) weak affinity, and (iii) comparatively low selectivity, hampering their real-world applications. The issue of non-biocompatible binding pH can be handled in four ways, i.e., the introduction of electron-withdrawing phenylboronic acids, such as carbonyl, sulfonyl, and fluoro on the phenyl ring 68, boronic acids comprising intramolecular tetracoordinate bonds B-N or B-O (Wulff-type), boronic acids with intramolecular coordinated bonds B-O (improved Wulff-type), and heterocyclic bonds 69. Since reversible condensation reactions are limited, the covalent interaction technique is less versatile. Besides, thermodynamic equilibrium is very difficult to achieve because strong covalent associations result in sluggish capturing and dissociation 70.

Non-covalent approaches use hydrophilic, hydrophobic 71, electrostatic, chelation interactions 72, and van der Waals forces to capture the template glycoproteins in the imprinting cavity. As MIPs are the “plastic” antibodies or antibody mimics, thus inspired by the natural antibodies' capturing mechanism, non-covalent interactions are more reliable for targeting glycoproteins. These approaches are more general and offer standard interactions for molecularly imprinted materials because of their utility, fast binding kinetics, and ease of template removal. However, minor disturbance in the interactions between the template and the monomer is sensitive. Since non-covalent interactions are typically weaker than covalent interactions, the selectivity of these materials with non-covalent interactions is generally not high. More precisely, boronate affinity-based covalent interactions are more suitable to immobilize the template glycosylated molecule on the surface of a substrate and non-covalent interactions are preferably developed in the following polymerization step with suitable functional monomers.

### 2.4 Imprinting approaches to develop MIPs

Usually, MIPs are fabricated by free radical polymerization in desired formats such as monoliths (bulk imprinting approach), microparticles, nanoparticles, thin films (surface imprinting), nanocomposites, etc. However, condensation polymerization is more suitable for surface imprinting due to controlled thickness and mild reaction conditions 73. The commonly used molecularly imprinted materials for glycoprotein and their characteristics have already been discussed in the previous section. Here, we will discuss the types of imprinting methods adopted to develop the glycoprotein imprinted nanoparticles for theranostic application.

#### 2.4.1 Bulk Imprinting

**Figure 2:**
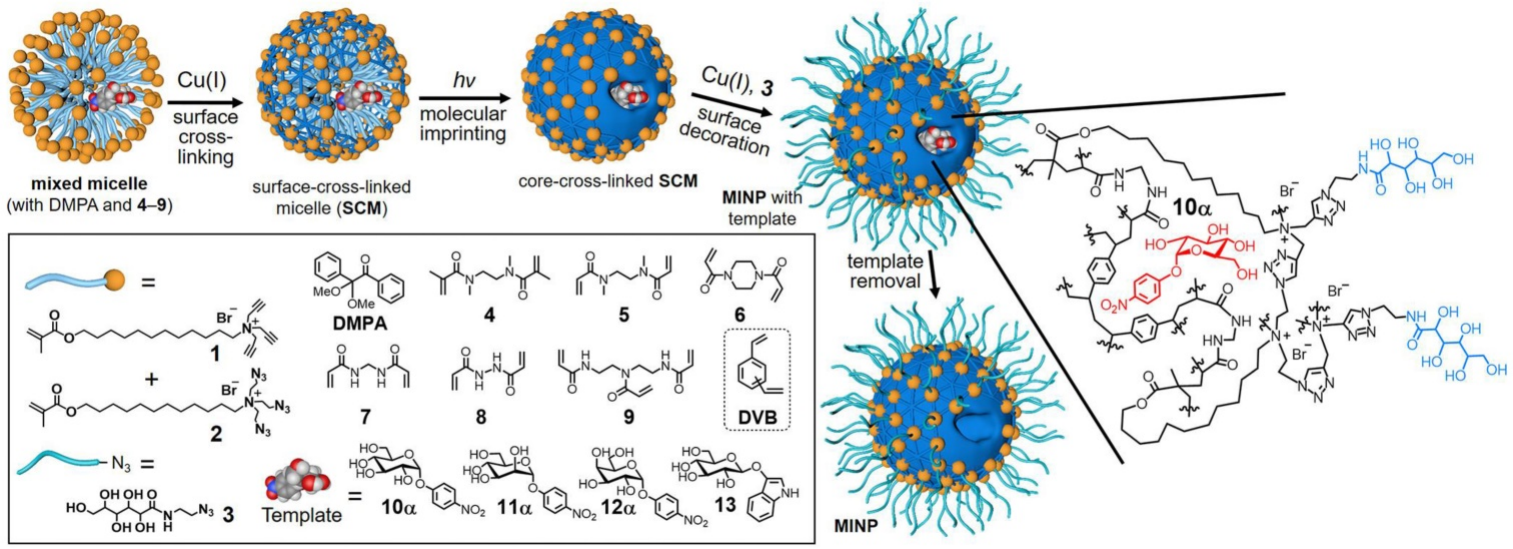
Preparation of glycan-binding MINP via molecular imprinting, with a schematic representation of the crosslinked structure using a mixture of DVB and 7 as the free radical cross-linkers. Adapted with permission from 57, copyright 2020 Nano Letters.

In bulk imprinting, the functional monomers, crosslinkers, initiators, and template molecules are mixed in the desired porogenic solvent, and polymerization is executed followed by crushing, grinding, and sieving to get the suitably sized particles. Then, the template is removed and obtained product is ready to use. This approach has clear advantages since it offers fast and facile synthesis, no need for complicated or expensive apparatus, and the imprinted materials are extremely pure. Liu and coworkers 39 reported a general and simple method to develop glycoprotein imprinted material via photolithographic approach using VPBA, which performed key roles, not only UV initiated polymerization to produce a thin layer imprinting array but also facilitated strong covalent affinity to capture the target glycoprotein. The facile synthesis via UV initiation takes less than 3 h for whole processing, has a high tolerance to interference, and is used in a wide range of pH. Lectins are a natural class of proteins, which capture carbohydrates with high specificity in physiological conditions as affinity tools for glycans but cannot deliver structural details about the glycoprotein's fragments 13. Considering the chemistry of lectins and the complexity of glycosylation, Zangiabadi et al. 57 developed a molecularly imprinted micelle via a convenient one-pot method, to specifically capture the target glycosides, glycans, and even the glycoproteins (Figure 2). In water, physiologically viable micromolar binding affinities are revealed, and small structural variations amongst glycans are identified. Among acrylate polymers, smart polymers using NIPAM as a monomer, have gained increasing attention since they change their characteristics like volume and hydrophilicity by modulating temperature, pH, or salt concentration, also have the potential to solve the existing issues in large-sized protein imprinting such as low mass transfer, low binding capacity and especially easy template removal as the pores are open by varying their environmental conditions. Xie et al. 74 proposed a quick and common molecular imprinting approach for specific capture of glycoprotein via dual pH-responsive polymer imprinted magnetic spheres using DMA as a pH-sensitive monomer along with VBA as an affinity monomer and MBA as a crosslinker containing OVA as template molecules.

Usually, the conditions needed for the imprinting of native proteins as a template may disturb the protein structure and cause difficulty in removing the protein template. To tackle this issue, Yang et al. 49 synthesized epitope-imprinted polyethersulfone (PES) polymer via PES phase inversion in a nonsolvent using epitope of TRF comprising of N-terminal sequence MRLAVGALL in dimethylacetamide solution. The phase inversion occurred when the mixture was transferred to the water by quick exchange of dimethylacetamide and water, the formation of TRF epitope imprinted solidified PES-particles appeared. Furthermore, due to an appropriate polarity of dimethylacetamide, it dissolved three different types of epitopes regarding their physical and chemical characteristics, so, the same strategy was used for the imprinting of three different epitopes, with a different GRAVY for the recognition of HAS, IgG, and TRF 75. It should be remembered, however, that there are also some significant disadvantages of bulk imprinting: 1) A lot of the recognition sites are positioned within the particles, leading to the consumption of a long time for saturation; 2) Captured targets are difficult to elute, causing poor recovery; 3) The efficiency of the template is limited, decreasing the binding potential and recognition capability of captured targets. The imprinting process can be updated in the ways to overcome these problems as follows: 1) By expanding the channel pores on the surface to allow a high mass transfer or using the smart polymers; 2) By reducing the size of polymer particles, including the nano-size, to enhance surface recognition sites. Some attempts have recently been made to fabricate nanoparticles via an updated phase inversion process 76, to benefit the future solution of the above-mentioned problems.

#### 2.4.2 Surface Imprinting

Surface imprinting is more advantageous, as the template may be perfectly oriented on solid support followed by the polymerization, which may give rise to a certainly aligned cavity, easily accessible to the rebinding of template molecules^77^. Compared to bulk imprinting, surface imprinting facilitates complete removal of the template and offers significant availability to target moieties, particularly for large peptides, proteins, and cells. The imprinted cavities in bulk-imprinted materials are usually incapable of binding these large molecules^78^. However, the controlled and appropriate thickness of the imprinting layer is very important in surface imprinting because the small thickness does not provide the desired specificity, and too large thickness buries the template inside the imprinting layer. A lot of surface imprinting strategies have been reported in the past few years, which described excellent efficiency for glycosylated molecules. Here, we have summarized some of the important and recent strategies. Pan and co-workers 46 developed a strategy based on enhanced binding affinity to specifically capture the glycoproteins via multiple boronic acid sites introducing polyethylene polyamine as a scaffold applying surface imprinting. Boronate affinity-based magnetic MIPs have low binding capacity because of less surface area due to the smoothness of silica-like polymers or magnetite surfaces and narrow working pH ranges offered by boronic acid ligands. Sun et al. 79 proposed a magnetic molecular imprinting approach by functionalizing porous TiO_2_, coated Fe_3_O_4_ with boronate affinity, followed by controlled polymerization of dopamine and named as Fe_3_O_4_@pTiO_2_@MIP. Compared to silica, flowerlike TiO_2_ coated on the Fe_3_O_4_ exhibits a high surface area and offers more binding sites and high binding capacity. Due to the strong electron-withdrawing effect of Ti (IV), Fe_3_O_4_@pTiO_2_@MIP selectively captured glycoproteins in a wide range of pH (6.0 to 9.0). Tian and co-workers 80 also explored a facile method using chelation interaction action for molecular imprinting of glycoproteins, where OVA was immobilized on the surface of MCM-48 via Cu^2+^-affinity chelation interactions, followed by the formation of an imprinting layer using PDA in a controlled manner. The prepared MIP exhibited fast binding kinetics and completely captured OVA within 30 min and showed the binding capacity of 395.30 mg g^-1^.

**Figure 3:**
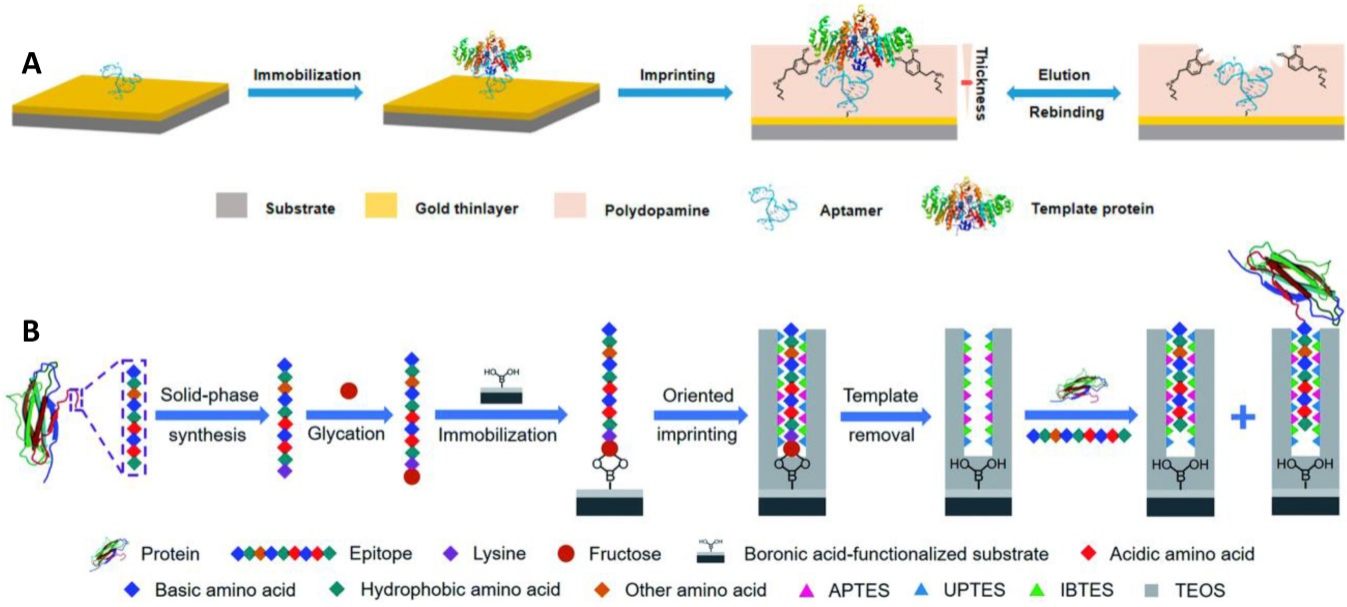
(A) Schematic illustration of the principle and procedure for preparing aptamer-MIP hybrid. (B) Schematic of the principle and procedure of controllable oriented surface imprinting of boronate affinity-anchored epitopes. Adapted with permission from 71, copyright, 2019 Analytical chemistry and 44, copyright 2019 Chemical science.

Aptamers have a lot of special advantages, including *in vitro* synthesis, facile modification, bio-compatibility, and a wide variety of possible targets 81. However, they usually stuck to poor affinity and low specificity due to their dynamic structures. The integration of MIPs and aptamers may produce ideal hybrid alternatives with desired characteristics. Li et al. 71 reported a novel approach based on the incorporation of aptamer and MIPs in the controlled and facile way for specific and high-affinity recognition of glycoproteins (Figure 3A). Thiol-functionalized aptamer, with less affinity and low specificity for a protein target (alkaline phosphatase, ALP), was fixed on the gold surface via S-Au bond and ALP was immobilized, followed by the controlled development of a thin imprinting layer via self-polymerization of DA. The resulting aptamer-MIP hybrid exhibited enhanced affinity and specificity towards ALP, cross-reactivity of 3.2-5.6%, and a dissociation constant of 1.5 nM. In the aforementioned strategies, the glycoproteins were used as a template, however, the template glycoproteins are unstable, adopted for the polymerization of the monomers for imprinting, and consequently, the imprinting cavity could specifically recognize the template glycoproteins, and importantly its characteristic fragments too. Bie et al. 59 developed lectin-like MIPs based on boronate-affinity glycan-oriented surface imprinting to tackle these issues. Glycans (multiple or single) were obtained by digesting target protein using glycosidase followed by purification via ultrafiltration and bound to the surface of the nano-magnetic cores using boronate affinity, imprinting layer was developed and glycan templates were removed. The resulting MIPs were capable of specifically capturing glycoproteins, their glycopeptides, and glycans as well. Moreover, MIPs exhibited an excellent IF value of 8.4 for RNase B and 21.8 for TRF with IE values of 44.8% and 43.5%, respectively. Another solution to the aforementioned problem is epitope imprinting 64, 65. However, epitope imprinting is stuck to epitope immobilization-related issues as mentioned above. Xing et al. 44 reported an epitope imprinting approach, where C-terminus epitope was glycated and attached to a boronic acid-modified core as a template, accompanied through controllable oriented surface imprinting, by reacting multiple silylating reagents and functionalities interacted with the epitope, such as APTES, UPTES, IBTES, and TEOS as depicted in Figure 3B. C-terminal nonapeptides for human β2-Microglobulin (B2M) and Mb were KIVKWDRDM and NYKELGFQG, respectively, selected from protein structure databases such as UniProt. The prepared epitope imprinting material exhibited Kd and Qmax value of (2.08 ±0.11) ×10^-7^ M and (169.03 ±5.50) nmol g^-1^, respectively, for β2-Microglobulin. Qin et al. 36 also developed a novel thermo-responsive dual template epitope imprinting approach, where C-terminal peptides of transferrin and human serum albumin were employed as templates and zinc acrylate as affinity monomer to produce a six-membered ring with epitope via metal chelation, NIPAAm as a thermos-responsive monomer, and EGDMA as a crosslinker for the polymerization which was executed in 30 min to generate the imprinting cavity.

## 3. Cancer diagnostics and imaging potential of glycoprotein imprinted nanoMIPs

Cancer must be diagnosed early to minimize death rates. Despite significant advancements in the area of improved diagnostic and prognostic tools, only a tiny percentage of tumor victims are identified in early stages. The identification of circulating tumor cells is an efficient method for predicting cancer prognosis at early stages 82. Unfortunately, due to the limited quantity of circulating tumor cells in circulating blood, effective techniques for their selective identification and separation are highly desired. Traditionally, antibodies have been in practice using immunoassays by specific recognition of the target, but the methodology suffers from massive cost, long time of fabrication, and instability 83. As artificial antibodies, MIPs are the effective alternative to natural antibodies, regarding their cost and stability, and have been effectively employed in the field of cancer diagnostics because of their captivating characteristics such as cost-effectiveness, high specificity, facile synthesis, high stability, and reusability. Additionally, MIPs show good compatibility with commonly used detection tools in sensor development. In addition, bioimaging is also one of the key foundations in biomedical study, since it enables early identification, surgical guiding, therapy impact monitoring, and clinical prognosis 84, 85. Conventionally, imaging modes include magnetic resonance imaging (MRI), X-ray, ultrasound, positron emission tomography (PET), and computed tomography (CT) 86, however nanoprobe-based fluorescence imaging is considered as one of the developing techniques, and it is growing rapidly in combination with nanotechnology for cancer research.

### 3.1. MIP-based biosensing technology for cancer diagnostics

NanoMIPs outperform natural antibodies in terms of thermal, physical, and chemical stability, giving biosensors a considerable advantage in identification even when exposed to environmental stresses 87. These nanoMIPs can be coupled with different types of detection systems to produce efficient biosensors for glycoproteins, however, glycoprotein imprinted MIPs-based sensors for tumor monitoring are discussed here. Ouyang et al. 88 reported a glycoprotein biomarker imprinted biofuel cell incorporating boronic acid anchored biliroxidase-carbon nanotube as a biocatalyst and signaling probe, and exhibited high sensitivity and specificity for AFP in human serum sample with a LOD value of 1 ng mL^-1^. Cuschieri and co-workers 89 used a nine-amino acid epitope of VEGF as a template followed by imprinting and resulting nanoMIPs were used to target human-VEGF. These nanoMIPs were integrated with quantum dots to make them fluorescent by embedding and surface attachment. The resulting material exhibited high affinity (Kd = 1.6 nM) for target human-VEGF and low affinity for non-target molecules. Although no definite imprinting parameters on the protein level were described, QD-MIPs directly targeted VEGF-overexpressed human melanoma cell xenografted in zebrafish embryos.

**Figure 4:**
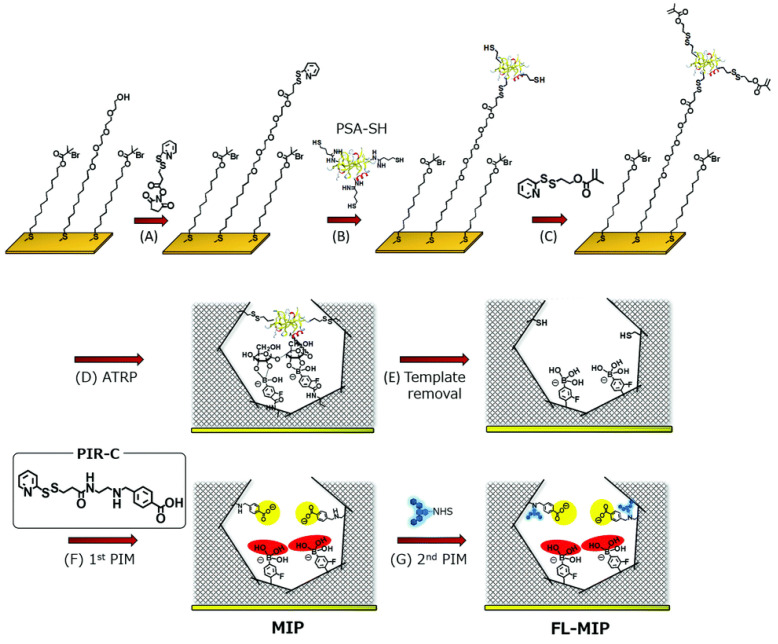
Synthesis procedure of molecularly-imprinted polymer (MIP) thin layer possessing orthogonal dual interaction sites and fluorescent reporting groups via molecular imprinting and sequential post-imprinting modifications (PIMs). Adapted with permission from 54, copyright, 2019 Journal of Materials Chemistry B.

For electrochemical sensing, the conductivity of the exposed surface is highly important to depict the strong response. Inspired by the electrical conductivity of PANI, Moreira, and Sales 90 reported an electrochemical sensor for CEA (a colorectal cancer biomarker). The integrated device consisted of a MIP-based sensor unit and a dye-sensitized solar cell as a voltaic cell. The imprinting layer was developed using CEA on a highly conducting PANI-counter electrode by electropolymerization and the template was removed using proteinase K action. The resulting sensor was assessed by electrochemical impedance spectroscopy and square-wave voltammetry as detection modes. The self-powered detection of CEA was similar to common biosensors with excellent sensitivity due to the incorporation of MIPs on the counter electrode. Irradiating the light on the surface of the photoanode of the dye-sensitized solar cell, a current was generated to run the device. Xing et al. 91 reported a dual MIP-based plasmonic immunosandwich assay for the sensitive sensing of the cancer glycoprotein marker. The technology was made up of two imprinted parts: Ag@SiO_2_ nanoparticles with an N-terminal epitope and a gold nanoparticle monolayer-coated glass slide with a C-terminal epitope. The strategy integrated high specificity of dual-MIPs and excellent sensitivity of SERS detection and evaluated for model glycoproteins and neuron-specific enolase (NSE) in human serum. Furthermore, relative to traditional ELISA tests, the MIP-PISA showed significant features, such as convenience, faster response, reduced sample amount, and a wider linear range. Zhou et al. also reported a similar dual MIP-based plasmonic immunosandwich assay comprising two imprinted parts where one of the parts is N-terminal or C-terminal (similar to the aforementioned study) of the polypeptide and the other part will be the glycosite, and other criterions are same 56. Saeki et al. 54 prepared PSA fluorescent sensor coupling molecular imprinting and following multistep post-imprinting modifications with a novel designed multifunctional reagent, fluorescent-signaling molecularly-imprinted nanocavities with orthogonal dual recognition sites for the sensing of prostate cancer biomarker glycoprotein, as shown in Figure 4. Zhang et al. 92 also presented a molecularly imprinted strategy coupled with a highly sensitive electrochemical detection system for the detection of PSA. The sensing substrate was made of MoS_2_ and gold nanoparticles, the surface imprinted cavities were made of PSA template and 4-mercaptophenylboronic acid, and the tracing tag was made of gold polymerized methylene blue composites labeled with 4-mercaptophenylboronic acid.

Tawfik et al. 93 developed a cost-effective, simple-to-use, and enzyme-free, cancer biomarker detection assay based on a new method involving fluorescent molecular imprinting conjugated polythiophenes (FMICPs). With a PLQY of up to 55 percent, the attractive conjugated polythiophenes structure offered a simple and inexpensive technique for free-enzyme signal production. Interestingly, the paper-printed probe was integrated with a low-cost smartphone and accessible prototype testing equipment for point-of-care cancer detection. In addition, the FMICP-nanofibers exhibited 80 times higher sensitivity than that of native FMICPs. For real sample applications, the proposed sensors were effectively used for the rapid detection of AFP in patients with liver cancer, with outcomes that were consistent with clinical ELISA results. Among these sensors, MIPs coupled electrochemical and fluorescent sensors are easy to miniaturize to develop a point-of-care device for rapid and on-site cancer testing just like the glucose sensors. Therefore, there is a need to develop these types of low-cost, easy-to-operate, highly sensitive POC devices, urgently. Some of the recent studies on biosensors for sensing of different types of glycoprotein cancer biomarkers and features of these materials are compared in Table 3.

### 3.2. MIPs-based imaging technology for cancer diagnostics

The capacity to track biomolecules and related compounds with excellent chronic and spatial resolution is crucial for understanding biological systems at the molecular level 93. Initially, it was started with the discovery of green fluorescent protein (GFP) and its derivatives contributed towards the progression of bioimaging 97 and evolved with the development of organic dyes 98 followed by the quantum dots 99 for both *in vitro* and *in vivo* studies. However, GFP and organic dyes-based imaging technologies are limited in sensitivity and long-term stability. In this regard, QDs are gaining increasing interest due to their splendid optical characteristics, dimension-dependent tunable emission wavelength, and consecutive excitation of QDs using a single light and wider spectral windows 100. Antibodies coupled with QDs have just been used to effectively monitor a broad variety of biological phenomena among the cells and animals, offering the site and morphology of cells 101. It revealed the process of tumorigenesis, growth, and metastasis by shedding light on the functioning of physiological processes.

**Figure 5:**
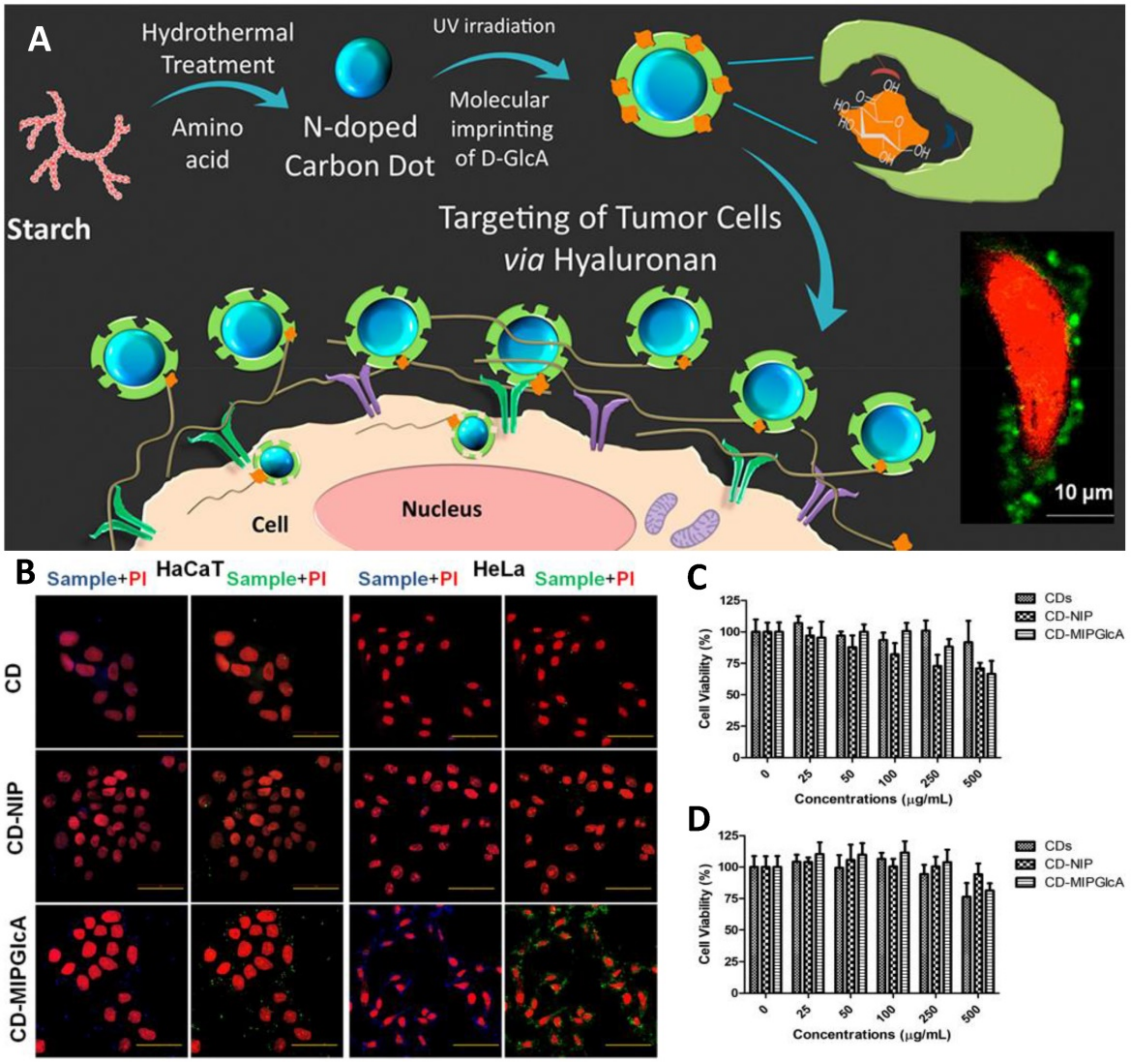
(A) Schematic diagram for tracking hyaluronan on cancer cell targeting and imaging by molecularly imprinted carbon dots; (B) Confocal microscope images of fixed HaCaT and HeLa cells treated with CDs, CD-NIP, and CD-MIPGlcA; Cell viability (MTT) assay of (C) HaCaT and (D) HeLa cells in the presence of CDs, CD-MIPGlcA, and CD-NIP for 24 h. Data are means from five different experiments. Adapted with permission from 105, copyright 2018 ACS Applied Materials & Interfaces.

Initially, small molecules such as nitrobenzoxadiazoles were coupled with nanoMIPs for developing fluorescent molecular imprinting probes for cell imaging 67. The surroundings of tumor tissue and the intracellular microenvironment both are complex, thus, enzymes there and ambient pH may result in quick fluorescence quenching 102. On the other hand, quantum dot-coupled imprinted particles provided better imaging performance. The imaging quality of QD-integrated MIPs is an alternative to immunofluorescent probes. Using QDs emissions, stimulated by UV light, Haupt's group suggested a unique local photopolymerization technique for producing MIP-based QDs (QDs@MIPs) for the identification of glucuronic acid and N-acetylneuraminic acid functionalized on the cells 103. Wang et al. 104 created several QD-based MIPs for cell pattern identification via multiple imaging by attaching to three different types of monosaccharides. Various fluorescence signals were detected, indicating the properties of these three monosaccharides on different cells, exposing their similarities and differences, and enabling the identification of particular cancer cells. Haupt's group then used MIPs with carbon nanodots (CD) to track hyaluronan on cancer cells. CDs, as opposed to QDs, exhibit more steady luminosity without blinking 105 as depicted in Figure 5. Furthermore, unlike QDs, they do not comprise heavy metals and are more biocompatible. Medina Rangel et al. 106 described a novel and effective approach based on CD-based MIPs, efficient for probing and staining hyaluronic acid existing on human epidermal cells, aided by a solid-phase method. Therefore, similar to polyclonal antibodies in probing and staining mechanism. Monosaccharide imprinted MIPs is not so specific to targeted sites, however, the epitope importing and glycoprotein imprinting technology is a more specific and direct approach and may lead to effective targeted imaging.

Epidermal growth factor receptors (EGFR), also commonly recognized as HER family, are expressed by the EGFR gene, and belong to tyrosine kinase receptors. Their overexpression may lead to the abnormal growth of the cell. Zhang et al. developed an imaging probe using carbon-dots (CDs) as a fluorescent-core followed by imprinting using the epitope imprinting technique 107. MIPs were prepared by reverse emulsion polymerization in a mixture of water/hexane combination in the presence of dioctyl sulfosuccinate sodium salt and Brij-30 as surfactants using an extracellular N-terminal nonapeptide epitope (EEKKVCQGT) modified with palmitic acid as a template. The function of palmitic acid was to keep the conformation of the epitope at the oil-water interface. The resulting imaging revealed that MIP-target HeLa cells (high-expression EGFR) glow more brightly than MIP-target MCF-7 cells (low-expression EGFR), indicating that the C-MIP targets tumor cells that overexpress EGFR. Due to its low tissue penetration, the single fluorescent imaging mode is not favorable for correct diagnostics. Considering the issue, Ren et al. 108 proposed glycan-oriented MIPs, using the BACOSI technique for high-resolution fluorescent and magnetic resonance imaging (MRI). First Gd-comprised fluorescent silica nanoparticles were fabricated and used as a core, followed by the functionalization of boronic acid to immobilize the epitope (Gal-NAc) of Tn antigen, and then surface imprinting was performed. Gd enhanced the fluorescent and MRI-imaging resolution as it is a well-known fact that doping of heteroatom (B, P) or metal ions in SiNPs can significantly boost the intrinsic properties 109, 110. Resulting NPs exhibited a size of 31.8 nm and revealed stable fluorescence capability with an adsorption capacity of 1.15 μM g^-1^, IF of 7.5, and low cytotoxicity. It has great potential for targeted cancer cell imaging with high resolution in real samples and MIPs integrated with imaging probes for targeted tumor imaging has been summarized in Table 4.

## 4. Image-guided cancer treatment potential of glycoprotein imprinted nanoMIPs

Cancer is the world's second-highest cause of death and a serious public health issue 113. Mainly, chemotherapy has been in practice for cancer treatment, however, it has some obvious drawbacks, such as being non-specific and harmful to healthy normal tissues and producing significant adverse effects. Unaddressed medical need is the development of an innovative way to address the aforementioned problem. Furthermore, tumor progression is usually occurred along with aberrant glycosylation, which gives rise to the possible markers for cancer cells. Alterations are often found during cancer, including abnormal branching of N-glycans, shortened O-linked chains, fucosylation, sialylation, and glycosphingolipid expression 114, 115. Several glycoproteins are tumor biomarkers linked to tumor cells. Therefore, glycoprotein targeted drug delivery is highly important, however, there are few ligands 12 that can identify or target important glycan biomarkers, limiting the establishment of glycan-targeting nanomedicine for cancer treatment.

### 4.1 NanoMIPs as a carrier for drug delivery

Among the advanced nanomaterials, molecularly imprinted polymers have numerous benefits of simple fabrication procedures, low cost, excellent stability, and facile incorporation with nanomaterials. Recently, their potential medical applications are found in the field of tumor targeting 116 and targeted drug delivery 117, 118. They are produced in novel formats and are a promising tool for cancer nanomedicines with a targeted drug release system. Premature drug release during blood circulation of chronically given cancer nanomedicines might limit the quantity of medication reaching tumors while also causing adverse effects 119, 120. Once the nanomedicines reach the target site, they intend to release the medicine in the required effective therapeutic amount. In addition, drug release kinetics are also very important and the antitumor drug should have a continuous stimuli-responsive. These problems may be tackled using nanoMIPs. Initially, non-covalent interactions were employed to capture the target monosaccharide of the glycoside but with the evolution of boronate affinity, nanoMIPs were capable to capture the template moieties with higher affinity, specificity, and pH-dependent detachment. For drug loading, non-covalent interactions are usually used to capture the drug molecules, tuned by pH or temperature according to the need, and hydrogen bonding is most common in practice. Because the terminal of glycoproteins and lipids on the cell membrane of cancer cells generally overexpress SA. Therefore, SA-imprinted mesoporous nanocarriers (SIMNs) exhibit cancer selectivity and targeted drug delivery. Considering the phenomenon, Yin et al. 121 fabricated a sialic acid-imprinted hydrogel layer on mesoporous silica-NPs to specifically bind the sialic acid moieties present on the glycoproteins and lipids attached to the cell membrane of cancer cells. The common pharmaceutical medication doxorubicin (DOX) was loaded in the mesopores of the silica-NPs and human hepatocellular carcinoma cells (HepG-2) were targeted for the drug release using sialic acid imprinted silica-NPs. These NPs targeted SA-overexpressed cancer cells and increased cancer cellular reception, ultimately resulting in better suppression of cancer cell growth.

**Figure 6:**
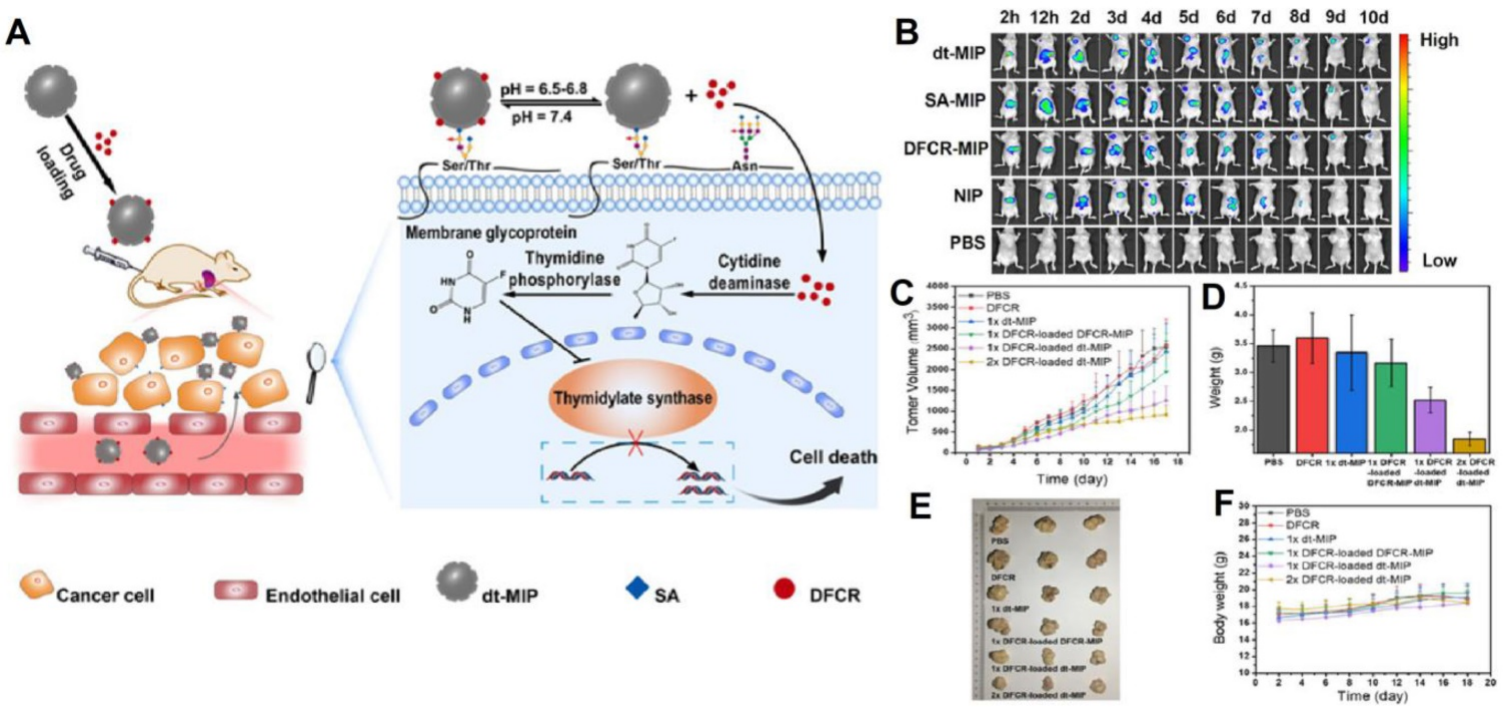
(A) Schematic of the drug transport and action mechanism in the dual-templated MIP-based smart prodrug delivery system, (B) *In vivo* fluorescence imaging of HepG-2 tumor (left upper chest) and liver site (upper abdomen) after intravenous injection ofNIR797-doped dt-MIP, SA-MIP, DFCR-MIP, NIP and PBS for different times, (C) Mean tumor volume, (D) Mean tumor weights after excision at 18 days, (E) Representative photographs of mice in different groups after treatment for 18 days, (F) Body weight of the mice in different groups at different time intervals. Adapted with permission from 116, copyright 2021 Angewandte Chemie.

Prodrugs are inert progenitors of effective therapeutic agents that are biologically converted or triggered in the body after delivery to enhance the parent drug's pharmacokinetic characteristics 122, 123, and more effective way as compared to direct chemotherapy. Liu and co-workers 116 integrated nanoMIPs with prodrug to produce a smart prodrug delivery probe, which exhibited longer retention, tumor microenvironment-triggered releasing, and specific targeting. 5'-deoxy-5-fluorocytidine (DFCR) was used as a prodrug, SA as a tumor marker, and this combination was used as a co-template, and the imprinting was done using a silica layer and resulted in a smart nano-MIP carrier. Prodrug-loaded smart carrier collected precisely and effectively at the tumor site before progressively releasing the drug (Figure 6). This MIP-based prodrug delivery is liver-independent instead of tumor-dependent, unlike other prodrug designs that frequently need in-liver bioconversion.

Using monosaccharide imprinted MIPs is not so specific to the targeted sites, however, the glycoprotein imprinted materials are more specific and direct and lead to the development of effective targeting nanomedicines. Considering this, Han et al. 124 fabricated CA125-imprinted graphene oxide, using DA as imprinting monomer revealing an IF of 6.4. While DOX was loaded on the CA125-imprinted GO, it exhibited enhanced cytotoxicity to the tumor cells and high specificity to the overexpressed CA125 sites. To prevent the conformational changes in the larger structure of glycoprotein, aqueous and compatible reactions conditions are necessary. Furthermore, the removal of proteins templates is difficult due to their large size and numerous interactions with different ligands which give rise to high cross-reactivity 125. As a result, the use of intact protein-imprinted MIPs for targeted delivery in cancer nanomedicine has been constrained.

Epitope-imprinting is an attractive way for the recognition of receptor glycoproteins on the cancer cells, where a short peptide is used as a template 126. The epitope-imprinting shows considerable benefits over whole protein-templated MIPs, including an increased affinity to target glycoproteins, decreased nonspecific bindings, simple template production, and broad synthesis conditions 127, 128. Based on these fascinating features, epitope imprinted NPs are extensively implemented for preparing nanoMIPs in tumor treatment. Hamid et al. 129 assessed HER2 receptors and produced the most effective epitope. The fabricated epitope and DOX were used as templates and PDA-based imprinting was executed on silica-NPs. The nanoMIP was directly used for ovarian cancer, indicated by increased DOX concentrations in tumor tissues, improved antitumor effectiveness, and longer mouse life.

Recently, metal-organic frameworks have gained increasing attention due to their well-ordered arrangement and unique porous structure with tunable porosity, especially when they are reduced in size to the nanoscale, they have become efficient drug carriers 130 and imaging probes 131 for delivering drugs, chemotherapy, and phototherapy 132. It is worth mentioning that zeolitic imidazolate framework-8 (ZIF-8) is biodegraded completely in an acidic environment. Considering this, Qin et al. 133 fabricated fluorescent zeolitic imidazolate framework-8 incubated it with DOX, as a core, and developed MIP using epitope of CD59 glycoprotein as a template, to specifically target MCF-7 cancer cells, termed as FZIF-8/DOX-MIP. This biodegradable FZIF-8/DOX-MIP can specifically target the CD59 cell membrane glycoprotein present on MCF-7 cancer cells. MIPs-layer was capable to be disrupted in the tumor acidic environment, allowing FZIF-8/DOX-core to further degradation in an acidic environment to release the DOX and demonstrated fluorescent imaging and active drug release. Relative to other cells, MCF-7 cells were more capable of phagocytosing FZIF-8/DOX-MIPs, and FZIF-8/DOX-MIPs were more deadly to MCF-7 cells.

### 4.2 NanoMIPs for photodynamic and photothermal therapy

**Figure 7:**
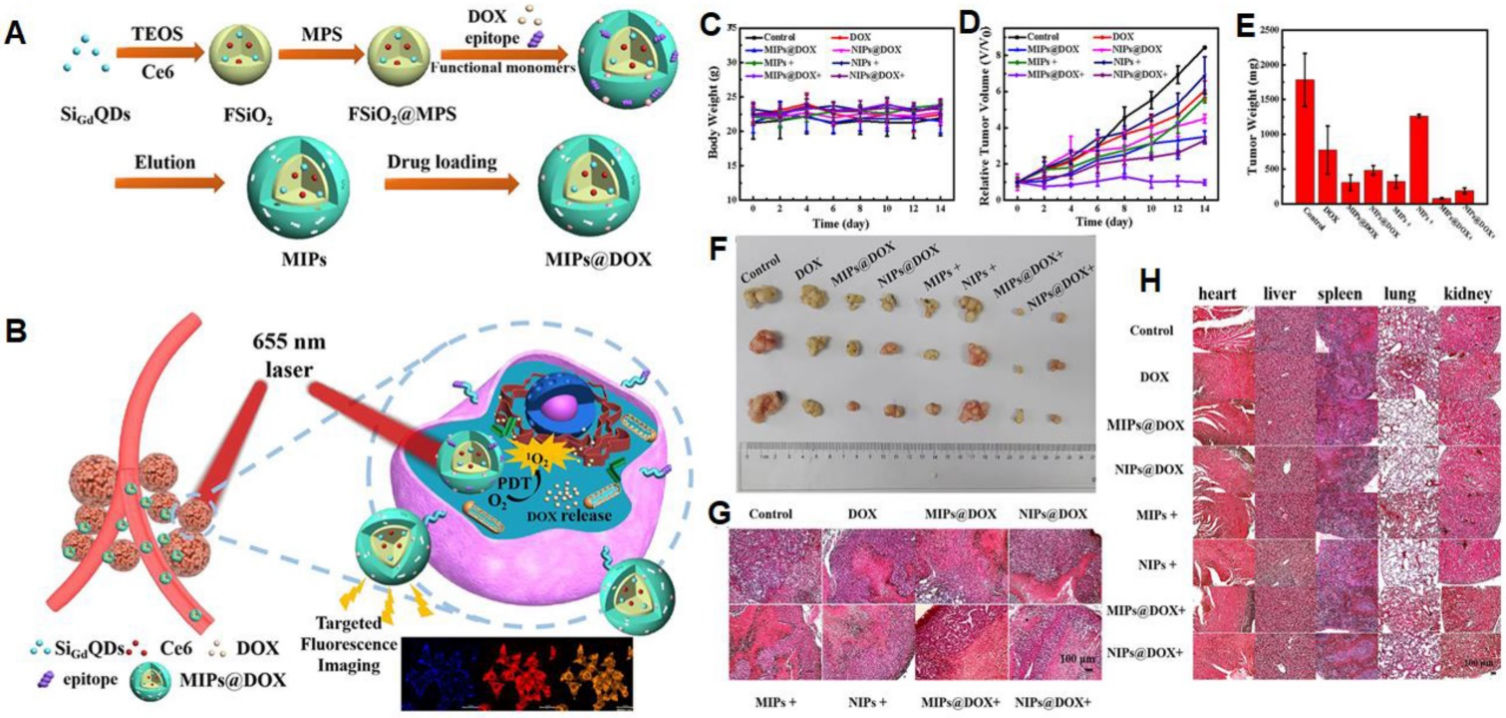
(A) Schematic Illustration for the Preparation of MIPs@DOX; (B) Schematic Illustration of MIPs@DOX for Targeted Chemo-Photodynamic Synergistic Treatment of Tumor *in vivo*. Body weight changes (C) and relative tumor volume (D) in 14 days after various treatments. The average tumor weight (E) and corresponding tumor tissues (F) after various treatments for 14 days. H&E staining of tumor sections (G) and main organs (heart, liver, spleen, lung, and kidney; H) were collected from various treatments groups on the 14th day. Adapted with permission from 135, copyright 2020 ACS Applied Materials & Interfaces.

NanoMIPs have potential applications in photodynamic and photothermal therapies for cancer treatment. In photodynamic therapy, a photosensitizer is introduced which produces the cytotoxic reactive oxygen species by the irradiation of light. These produced reactive oxygen species kill targeted cells at a particular irradiated site. Liu et al. 134 described an inducible epitope imprinting approach where an imprinting template, a folate receptor-a (FR-a) epitope (QTRIAWARTELLNVAMNAKH) was folded to an a-helix shape and used as a template to produce nanoMIPs to capture the target peptide alter it from disordered-to-ordered conformational change, consequently inducing binding sites in the target receptors. To make the MIPs more specific to the target, strong epitopes are created by obtaining the sequence and 3-dimensional arrangement from data banks and identifying epitopes using in silico techniques. In comparison to NIP, MIPs show enhanced binding affinity to FITC-modified, peptide, and recombinant FR in fluorescence polarization assays. For fluorescence imaging and flow cytometry, polymers tagged with the fluorescent dye Nile red demonstrated stronger binding with human cervical cancer HeLa cells (FR- positive) than with lung cancer A549 (low FR) cells. Compared to NIP-NPs and NPs imprinted with the twisted peptide, MIP-NPs encapsulate the near-infrared dye IR-783 accumulated at tumor sites of animals transplanted with HeLa cells. Methylene blue (MB) was incorporated in the pre-polymerization mixture as a photosensitizer to develop nanoMIPs with a size of 78 nm for the generation of reactive oxygen species and these nanoMIPs demonstrated a strong inhibition effect on HeLa cancer xenografts in the following photodynamic treatment. In another study, Zhang et al. 63 used an N-terminal α-helix of the p32, an overexpressed glycoprotein on the tumor cell membrane that was grafted on apamin substrate, as a template. As a result, apamin sites were substituted with topologically comparable residues from p32's N-terminal -helix, however, the residues with the highest -helix affinity were preserved. The peptide was linked with palmitic acid at the free amino group of the side - chain to guarantee that it is well-orientated at the contact of the water and oil regions throughout polymerization. Microemulsion polymerization was carried out at room temperature to produce sub-40 nm-sized nanoparticles that were specifically bound to P32, P32-up regulated cancer cells, and effectively performed the targeted photodynamic treatment *in vivo*. Recently, Peng et al. 135 demonstrated dual template nanoMIPs revealing two different therapies, simultaneously as shown in Figure 7. The silica nanoparticles were used as a core, comprising fluorescent gadolinium-silicon quantum dots for fluorescence imaging and MR-imaging, and photosensitizers (Ce6) for liberating toxic O_2_ by 655 nm laser radiations to kill the tumor cells. The polymerization was done in the presence of two templates, the epitope of CD59 (YNCPNPTADCK) and DOX, and after the removal of the template, the resulting nanoMIPs were capable to specifically load the drug and target the CD59-overexpressed tumor sites. *In vitro* and *in vivo* experimentations revealed, after specific targeting the CD59-overexpressed tumor sites, nanoMIPs effectively released DOX in acidic microenvironment and irradiating 655 nm laser radiations, photosensitizers (Ce6) killed the tumor cells and allowed clear fluorescent and MR-imaging.

Photothermal therapy is frequently used in cancer care since it promotes the apoptotic or necrotic behavior of cancer cells while also suppressing tumor development by creating a localized thermal effect 136. Yin et al. 137 reported sialic acid imprinted nanoMIPs for targeted photothermal tumor therapy. The nanoMIPs were fabricated by immobilizing sialic acid template on boronic acid-functionalized AuNRs followed by controlled imprinting. HepG-2 as overexpressing SA cells and L-02 as normal cells were treated with FITC-doped MIP nanorods to differentiate cancer cells from normal cells, and confocal microscopy and flow cytometry demonstrated that these nanoMIPs efficiently target the HepG-2 cells. *In vitro* investigations revealed that after 6 minutes of illumination with a 750 nm laser beam, MIP nanorods caused apoptosis in HepG-2 cells as measured by the MTT assay, however, the survival of L- 02 cells maintained greater than 85%. For *in vivo* studies, nanoMIPs demonstrated a substantial decrease in tumor volume in 2 weeks after laser irradiation and a strong photothermal impact on the ablation of HepG-2 tumors in mice. AuNR-MIPs might be used as selective medicine for tumor ablation employing photothermal treatment without harming normal tissue.

### 4.3 NanoMIPs themselves as therapeutics

Human epidermal growth factor receptor-2 (HER2) belongs to the epidermal growth factor receptor (EGFR) class and is upregulated in 20-30% of breast cancers 138, 139. The most powerful EGFR signaling dimer among the heterodimers is HER2/HER3 dimer 140. Prevention of HER2 dimerization with other EGFRs, particularly HER3, offers an appropriate therapy for HER2-positive breast cancer. Dong et al. 141 presented a novel methodology incorporating molecular imprinting technique for the inhibition of HER2+ breast cancer growth. The glycan-imprinted nanoparticles were prepared using HER2 N-glycans as a template prepared through the digestion of HER2 protein by PNGase F, immobilized on the fluorescent silica nanoparticles via boronate affinity followed by the imprinting employing a cutting-edge imprinting technique known as boronate-affinity-controllable oriented surface imprinting. The fabricated nanoMIPs may capture nearly all HER2 glycans and prevent HER2 dimerization to other HER2 receptors, limiting downstream signaling pathways and reducing the development of HER2+ breast cancer. The in-vitro studies revealed that nanoMIPs attached to HER2+ cells specifically, thereby inhibiting cell proliferation up to 30%. *In vivo* experimentation demonstrated that nanoMIP-treated group's average tumor volume was only approximately half that of non-treated groups. The study offers a novel way to treat HER2-positive breast cancer and adds to the growing body of data that nanoMIPs may be used to treat cancer. Among the techniques, the pristine protein imprinted polymers are difficult to manufacture, although the procedures are basic and straightforward for producing nanoMIP. This is especially true when the proteins are unstable or not readily available in their pure condition. Although epitope-imprinting is an effective way to tackle the issue, but still needs further improvement in the conformational preservation of epitope peptide throughout the imprinting process.

**Figure 8:**
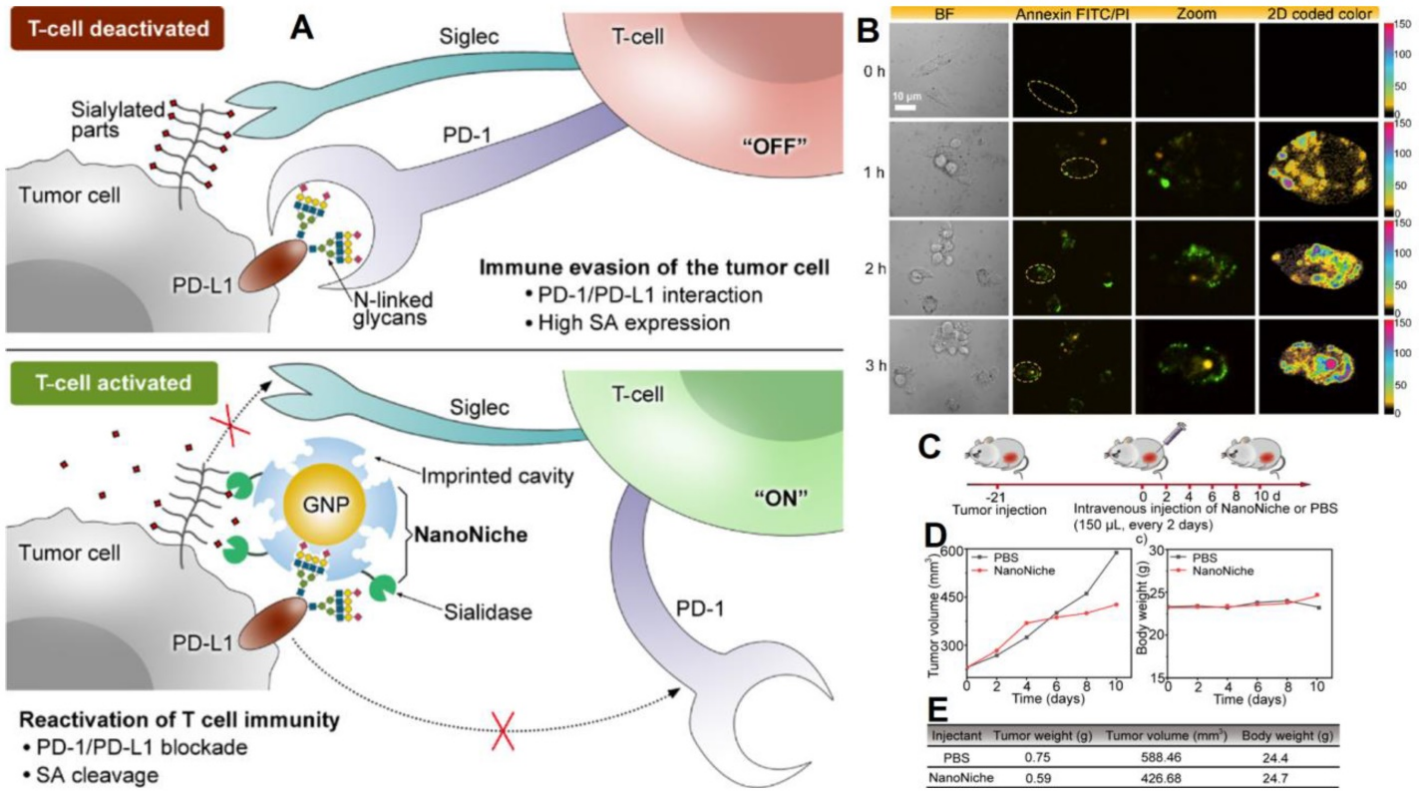
(A) The principle of tumor cell immune evasion from T-cells and the reactivation of T-cell immunity by blocking PD-L1 through N-glycan-imprinted NanoNiche with a desialylation function schematic illustration; (B) Microscopy images showing the T-cell-mediated tumor cell-killing effect in MDA-MB-231 cells incubated with NanoNiche (200 μL) after treatment for 0, 1, 2, and 3 h; Schedule of various treatments in tumor-bearing mice; (C) Average tumor volumes after various treatments at different intervals of time; (D) Body weights of mice in various groups after treatments at various time intervals; (E) *In vivo* anticancer activity outcomes of mice in various groups after a 10-day therapy. Adapted with permission from 147, copyright 2021 ACS Applied Bio Materials.

Discovering of programmed death 1 (PD-1) and programmed death-ligand 1 (PD-L1) signaling pathways have altered clinical cancer therapy since their inhibition plays a key role in the regulation of immunological T-cells 142-144. Inhibiting PD-1 or PD-L1 to reactivate T-cell response has proven an effective cancer therapy technique, and antibodies targeting PD-1 or PD-L1 have been extensively applied for the treatment of many malignancies 145, 146. However, N-linked glycosylation of PD-L1 can make its antigenic epitopes unidentifiable by traditional PD-L1 antibodies. Zhou et al. presented “NanoNiche”, a synthetic PD-L1 antibody integrated with desialylation functioning to boost up immune cell activation 147, as shown in Figure 8. NanoNiche was prepared by immobilizing the glycans of PD-L1 on gold nanoparticles followed by the formation of a silica imprinting layer. Interestingly, sialidase was covalently attached to the nanoMIPs for desialylation on the surface of the cell. NanoNiche has been shown to target the PD-L1 on the surface of tumor cells, causing desialylation and inhibiting the proliferation of MDA-MB-231 cells *in vitro*. *In vivo* studies in tumor cells reveal improved treatment effectiveness. As a result, the NanoNiche-sialidase conjugate is a viable immune-checkpoint blocking treatment option. Some of the recent studies depicting the current cancer treatment, targeting specific cells with different mechanisms of treatment and features of these materials are compared in Table 5.

## 4. Conclusion and Future Perspectives

Recognizing both diverse functions of glycoproteins in different physiological processes and natural mechanisms, a lot of glycoproteins imprinted approaches have been engineered or updated to meet the standards of targeted proteomics analysis. MIPs are widely used for generating antibody-like substitutes that have allowed complex samples to specifically enrich target glycoproteins. Because of their outstanding targeting capability, biocompatibility, high specificity, excellent anti-interference performance, and pH-controlled binding/dissociation, they have shown promising applications in proteome analysis, sensing, disease diagnostics, tissue engineering, and drug delivery. To facilitate its intensified use, much advancement is needed in glycoprotein imprinting. Regarding the prospects for glycoprotein imprinting, there are certain substantial aspects such as dimensions of imprinting material, complexity, the fragility of the structure of glycoproteins, difference in solubility of monomers, and template molecule, purity, and availability of a template, and uncontrolled thickness of imprinting layer. The size of imprinting material and overall particle should be reduced and have a flexible network for facile removal of template molecules and *in-vivo* applications. In addition, novel smart temperature-responsive or polymer-materials should be considered for facile template removal and fast extraction/desorption kinetics. Due to the complex structure of glycoprotein, the selection of monomers and the mechanism of interaction should be well studied, not only on an experimental basis but also by theoretical simulations. Moreover, for the specific extraction of diverse glycoproteins, novel ligands such as aptamers could be valuable options. Furthermore, glycoproteins are water-soluble but their monomers are limited, which need to be developed. On the other hand, using different types of molecular structural modeling programs such as the Gaussian program can facilitate the Gauss View for molecular imprinting of glycoproteins.

Another barrier is the uncontrolled thickness of the imprinting layer and at present, a limited number of biocompatible and self-polymerizable ligands are available for imprinting of glycoproteins. Among these polymer materials, time-dependent controllability of self-polymerization has been revealed by dopamine/APBA and TEOS. This challenge can be addressed by finding more effective functional monomers or changing the current monomers with unique functionalities or structures. Moreover, ATRP and RAFT polymerization methods are also useful for controlling the thickness of the imprinting layer. PolyNIPAM is approved by FDA due to its biocompatibility and other polyacrylamides are less toxic revealed by MTT assay in most of the living cells. For practical applications, MIPs should have monoclonal antibody-like features such as high affinity, fast recognition kinetics, and high specificity. An effective nanoMIP-imaging probe for clinical usage is biodegradable or quickly eliminated, with minimal toxicity and a strong imaging response. The imprinted particles should have a diameter less than 8 nm to be excreted by kidneys otherwise larger-sized particles are cleared by the liver. As the renal clearance is ideal due to rapid response, but size-controlled development at such a small scale is challenging. Owing to their advantages, nanoMIPs also have potent cancer therapeutic characteristics and revealed excellent performance in precise targeting, a systemic circulation, and controlled drug delivery systems. NanoMIPs can also combine several functionalities into a single system, making them a potent and adaptable substrate for complementary medications to cure cancers. For real-world cancer therapeutic applications and future clinical translation, multifunctional MIP-based nanomedicines are urgently required, considering their biocompatibility, specific targeting, controlled drug release kinetics, excellent clear imaging, and relevant animal models for *in vivo* studies, which must be evaluated and explored for future clinical translation. Regarding blood compatibility, their effect on blood coagulation and inflammatory reaction must also be evaluated.

## Figures and Tables

**Table 1:** Types and functions of functional monomers in glycoprotein imprinting.

Types of monomers	Name	Structure	Functions	References
Acrylate Monomers	Acrylic acid	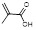	Chelator	33
	Vinylbenzyl iminodiacetic acid	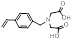	Strong chelator	34
	Methacryl amide	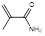	Impart hydrophilicity	16
	Aminoethyl methacrylate	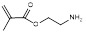	Impart hydrophilicity	35
	N-isopropylacrylamide	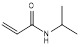	Impart thermo-responsive properties	36
	2-(Dimethylamino) ethyl methacrylate	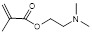	Impart pH-responsive properties	37
	(3-acrylamidopropyl) trimethyl ammonium chloride	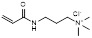	Anion exchanger	35
	N-phenylacrylamide	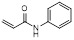	π-π interaction facilitator	34
	N, N'-methylene bis(acrylamide)	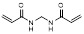	Hydrophilic crosslinker	38
	4-Vinylphenylboronic acid	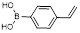	Capture cis-diols	39
	2,4-Difluoro-3-formylphenylboronic acid	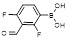	Capture cis-diols at reduced binding pH	40
	6-Vinylbenzoxaborole	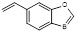	Capture cis-diols at reduced binding pH (improved Wulff affinity)	41
Silica Monomers	Tetraethyl orthosilicate	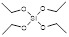	Initial silica precursor	42
	3-Aminopropyltriethoxysilane	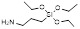	Facilitate amine-group for functionalization	43
	N-octyltrimethoxysilane		Facilitate hydrophobicity	43
	3-Ureidopropyl-triethoxysilane	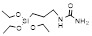	Two-fold cyclic hydrogen bond donor	44
	3-Methacryloyloxypropyl trimethoxysilane	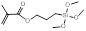	Coupling silica with an acrylate polymer	38
Polydopamine monomers	Dopamine	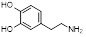	Facilitate coating on any hydrophilic or hydrophobic surface	45
	4-aminophenyl boronic acid	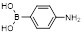	Capture cis-diols	40
Polyaniline monomers	Aniline		Basic hydrophobic polyaniline precursor	46

**Table 2:** Summary of important features of aforementioned molecularly imprinted materials for glycoproteins

Imprinting materials	Facile synthesis	Time controlled thickness	Availability of functional monomers	Compatibility with boronate affinity	Flexibility or easy template removal
Silica	+++	+++	++	+++	++
Polydopamine	+++	+++	+	+++	++
Polyacrylate	+	+	+++	++	+
Smart Polyacrylate	+	+	+++	++	+++
Polyaniline	+	+	+	++	+
Polyether Sulphone	+++	+	+	+	+

The accessibility is measured by the quantity of “**+**” symbols.

**Table 3:** Summary of MIPs-coupled biosensors for detection of different types of glycoprotein cancer biomarkers.

Sensor type	Monomers	Imprinting type	Target protein	LOD	Cancer type	Linear dynamic range	Sample	Reference
Electrochemical	ANI	Bulk imprinting	CEA	0.10 pg mL^-1^	Colorectal cancer	0.025 ng mL^-1^	Human urine	94
	APBA/o-PD	Surface imprinting	PSA	0.03 pg mL^-1^	Prostate cancer	1.0×10^-4^ to 1.0×10^4^ ng·mL^-1^		92
	Phenol		CA 125	0.5 U mL^-1^	Ovarian cancer	0.5-400 U mL^-1^		95
Fluorescence	FMB		PSA	2.35 ng mL^-1^	Prostate cancer	5 to 150 ng mL^-1^		54
	PYM, MPC/MBAA		AFP	0.27 ng mL^-1^	HCC	-		96
SERS	APTES, UPTES, IBTES, TEOS		CEA	5.6 × 10^-14^ M	Colon cancer	1 ng mL^-1^ -10 μg mL^-1^	Human serum	56
			NSE	10 pg mL^-1^	small cell lung cancer	100 pg mL^-1^ - 10 μg mL^-1^		91

**Table 4:** Summary of MIPs-coupled imaging technology for cancer diagnostics.

Imaging type	Template	Monomers	Tumor model	Imaging probe	Cancer type	Reference
Fluorescence	Epitopes of EGFR	Aam/MBAA	HeLa cells (over-expression EGFR), HeLa-tumor-bearing mice	CDs	Cervical cancer	107
	Glucuronic acid	AB, MAM/EGDMA	HA on HaCaT cells	N-doped CDs		105
	Hyaluronan	NIPAM/EbAm		Rh B		111
Fluorescence & MRI	Gal-NAc	TEOS	MCF-7 cells	Gd-doped SiNPs	Breast cancer	108
SERS	Sialic acid		HepG-2	Raman-active AgNPs	HCC	112

**Table 5:** NanoMIPs executes multitasks for cancer therapy targeting specific sites.

Type of therapy	Template	Monomers	Tumor model	Drug	Targeted cancer	Reference
Drug delivery	CA125	DA	SMMC-7721	DOX	Liver cancer	124
	SA	NAPMAAm, NIPAAm, Aam/MBAA	SA over expressed HepG-2 cells			121
	N-terminal epitope of CD59 glycoprotein	TFMA, DMAEMA, NIPAAm, TBAm/ BAC	MCF-7 cancer cells, MCF-7-tumor-bearing mice		Lung cancer, breast cancer	133
	Linear peptide of HER2	Aam, ZnA/ EGDMA	SK-BR-3 cells		HER2- breast cancer	148
	SA	TEOS	HepG-2, MCF-7 cells, HepG-2 tumor-bearing mice	DFCR (prodrug)	Hepatic cancer & breast cancer	116
Photodynamic therapy	N-terminal peptide of FR-α	TFMA, Aam/MBAA	HeLa cells (FR-α over-expressed cells), HeLa-tumor-bearing mice	MB (photosensitizer)	Cervical cancer	134
	Disulfide-linked α-helix-containing peptide-apamin	Aam/ MBAA	4T1 murine cells and BxpC-3 human cells, 4T1tumor-bearing mice		Breast cancer	63
Photodynamic therapy + Drug delivery	Linear peptides of CD59 DOX	NIPAAm, TBAm, Aam/ MBAA	MCF-7 cancer cells	DOX + photosensitizers Ce6 liberating ^1^O_2_		135
Photothermal therapy	SA	TEOS	HepG-2, HepG-2 tumor-bearing mice	AuNRs	Liver cancer	137
Inhibition of HER2 breast cancer	N-glycans of HER2 glycoprotein		SKBR-3, SKBR-3 tumor-bearing mice	NanoMIPs themselves as therapeutic	HER2- breast cancer	141
Immune checkpoint blockade therapy	N-glycans of PD-L1 glycoprotein		MDA-MB-231, MDA-MB-231 tumor-bearing mice	NanoNiche itself as therapeutic	Human breast cancer	147

## Abbreviations

Am: acrylamide
AFP: α-fetoprotein
APBA: 3-acrylamidophenyl boronic acid
APTES: 3-aminopropyltriethoxysilane
APTMS: 3-aminopropyl trimethoxysilane
BAC: N, N'-diacrylylcystamine
BACOSI: boronate affinity controllable oriented surface imprinting
CD: circular dichroism
CEA: carcinoembryonic antigen
DMA: 2-(dimethylamino) ethyl methacrylate
DMAEMA: dimethylaminoethyl methacrylate
EGDMA: ethylene glycol dimethacrylate
FITC: fluorescein isothiocyanate isomer I
FM: 4-[2-(N-methacrylamido) ethylaminomethyl] benzoic Acid
FMB: 4-(2-methacrylamidoethylcarbamoyl)-3-fluorophenylboronic
GRAVY: grand average of hydropathicity
HEMA: 2-hydroxyethylmethacrylate
HSA: human serum albumin
IBTES: isobutyltriethoxysilane
IE: imprinting efficiency
IF: imprinting factor
MAA: methacrylic acid
Mb: myoglobin
MB: methylene blue
MBA: N, N′-methylenebisacrylamide
MIPs: molecularly imprinted polymers
MNPs: magnetic nanoparticles
MPC: 2-methacryloyloxyethyl phosphorylcholine
MPTMS: 3-methacryloyloxypropyl trimethoxysilane
NAPMAAm: N-(3-aminopropyl) methacrylamide hydrochloride
NIPAAm: N-isopropylacrylamide
NPs: nanoparticles
o-PD: o-phenylene- diamine
OVA: ovalbumin
PISA: plasmonic immunosandwich assay
PSA: prostate-specific antigen
RNase B: ribonuclease B
SELEX: systematic evolution of ligands by exponential enrichment
SERS: surface-enhanced Raman scattering
TBAm: N-tert-butyl acrylamide
TFMA: 2-(trifluoromethyl) acrylic acid
TRF: transferrin
UPTES: 3-ureidopropyl-triethoxysilane
VEGF: vascular endothelial growth factor
VPBA: 4-vinylphenylboronic acid
ZnA: zinc acrylate
